# Supplementary material for: Single-cell transcriptome maps of myeloid blood cell lineages in Drosophila
Source: Nat Commun. 2020 Sep 8;11:4483. doi: 10.1038/s41467-020-18135-y (PMC7479620; doi:10.1038/s41467-020-18135-y)
Supplement: Supplementary file 1 — Supplementary Information [file 41467_2020_18135_MOESM1_ESM.pdf]

Supplementary Information

Single-cell transcriptome maps of myeloid blood cell lineages in *Drosophila*

Bumsik Cho[1#], Sang-Ho Yoon[1#], Daewon Lee[1], Ferdinand Koranteng[1], Sudhir Gopal Tattikota[2], Nuri Cha[1], Mingyu Shin[1], Hobin Do[1], Yanhui Hu[2], Sue Young Oh[3], Daehan Lee[1], A. Vipin Menon[1], Seok Jun Moon[3], Norbert Perrimon[2,4], Jin-Wu Nam[1,5,6*] and Jiwon Shim[1,5,6*]

[1]Department of Life Sciences, College of Natural Science, Hanyang University, Seoul 04736, Republic of Korea

[2]Department of Genetics, Harvard Medical School, Boston, MA 02115, USA

[3]Department of Oral Biology, Yonsei University, College of Dentistry, Seoul 03722, Republic of Korea

[4]Howard Hughes Medical Institute, Boston, MA 02115, USA

[5]Research Institute for Natural Sciences, Hanyang University, Seoul 04736, Republic of Korea

[6]Research Institute for Convergence of Basic Sciences, Hanyang University, Seoul 04736, Republic of Korea

[#]These authors contributed equally to the work

*Corresponding authors: jshim@hanyang.ac.kr; jwnam@hanyang.ac.kr

Supplementary Figure

Supplementary Table

Supplementary Figure 1

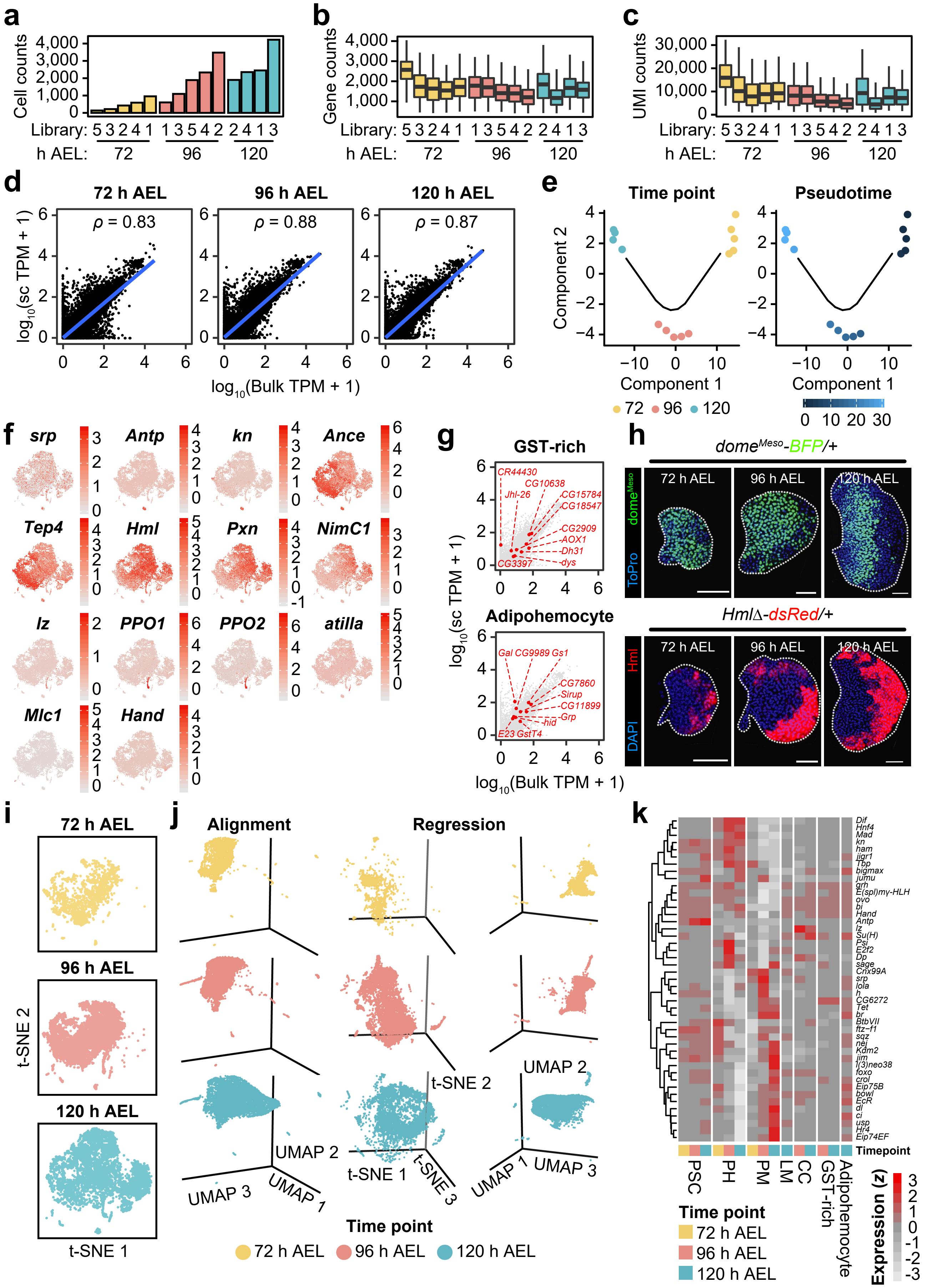

Supplementary Fig 1. scRNA-seq of *Drosophila* lymph glands

a. Cell counts per each sequencing library are colored by sampling timepoints. Libraries are ordered by the number of cells (72 h, yellow; 96 h, pink; 120 h, blue). At 72 h AEL, prohemocytes establish the medullary zone, a subset of which initiates differentiation into mature hemocytes. The medullary zone and the cortical zone together with the intermediate zone in between the two are observed at 96 h AEL. The lymph gland prohemocytes continues to differentiate afterward and the cortical zone expands by 120 h AEL.

b-c. Box plots of gene (**b**) or UMI (**c**) counts per sequencing library colored by sampling timepoints (72 h, yellow; 96 h, pink; 120 h, blue). Libraries are ordered as in (**a**). For each boxplot, the center line represents the median. Upper and lower limits of each box represent the 75th and 25th percentiles, respectively. The whiskers represent the highest and the lowest data points still within size of 1.5× inter-quartile range.

d. Spearman correlation analyses between pseudo-bulk scRNA-seq and matched bulk RNA-seq samples (≥ 1 Transcripts Per Million (TPM); 8,724, 7,654, and 7,627 genes at 72, 96, and 120 h AEL, respectively). Expression values are transformed to the $\log_{10}$ scale.

e. Trajectory analyses of pseudo-bulk scRNA-seq libraries using Monocle 2 colored by each timepoint (left; 72 h, yellow; 96 h, pink; 120 h, blue) or pseudotime (right) (72 h, yellow; 96 h, pink; 120 h, blue). Color bar indicates pseudotime.

f. Expressions of known marker genes in the major cell types. Color bar indicates the level of scaled gene expression.

g. Expressions of top 10 signature genes of GST-rich (top) or adipohemocyte (bottom) in pseudo-bulk scRNA-seq and matched bulk RNA-seq. Expression values from three timepoints in each dataset were averaged and transformed to the $\log_{10}$ scale.

h. Expressions of *dome*^{Meso}-positive prohemocytes (green, *dome*^{Meso}; blue, DAPI) or *Hml*-positive plasmatocytes (red, *Hml*; blue, DAPI) at 72, 96, or 120h after egg laying (AEL). White scale bar indicates 30μm. Lymph glands are demarcated by white dotted lines.

i. Separation of integrated *t*-SNE projection (related to Fig. 1**c**) of wild-type lymph glands by timepoints.

j. Three-dimensional UMAP projection of aligned hemocytes from the lymph gland (left). Three-dimensional *t*-SNE (middle) and UMAP (right) plots of lymph gland hemocytes normalized using regression.

k. Heatmap presentation of transcription factors in the lymph gland analyzed by SCENIC. Annotations at the bottom indicate timepoints and major cell types (72 h, yellow; 96 h, pink; 120 h, blue). Color bar indicates the level of scaled gene expression.

Supplementary Figure 2

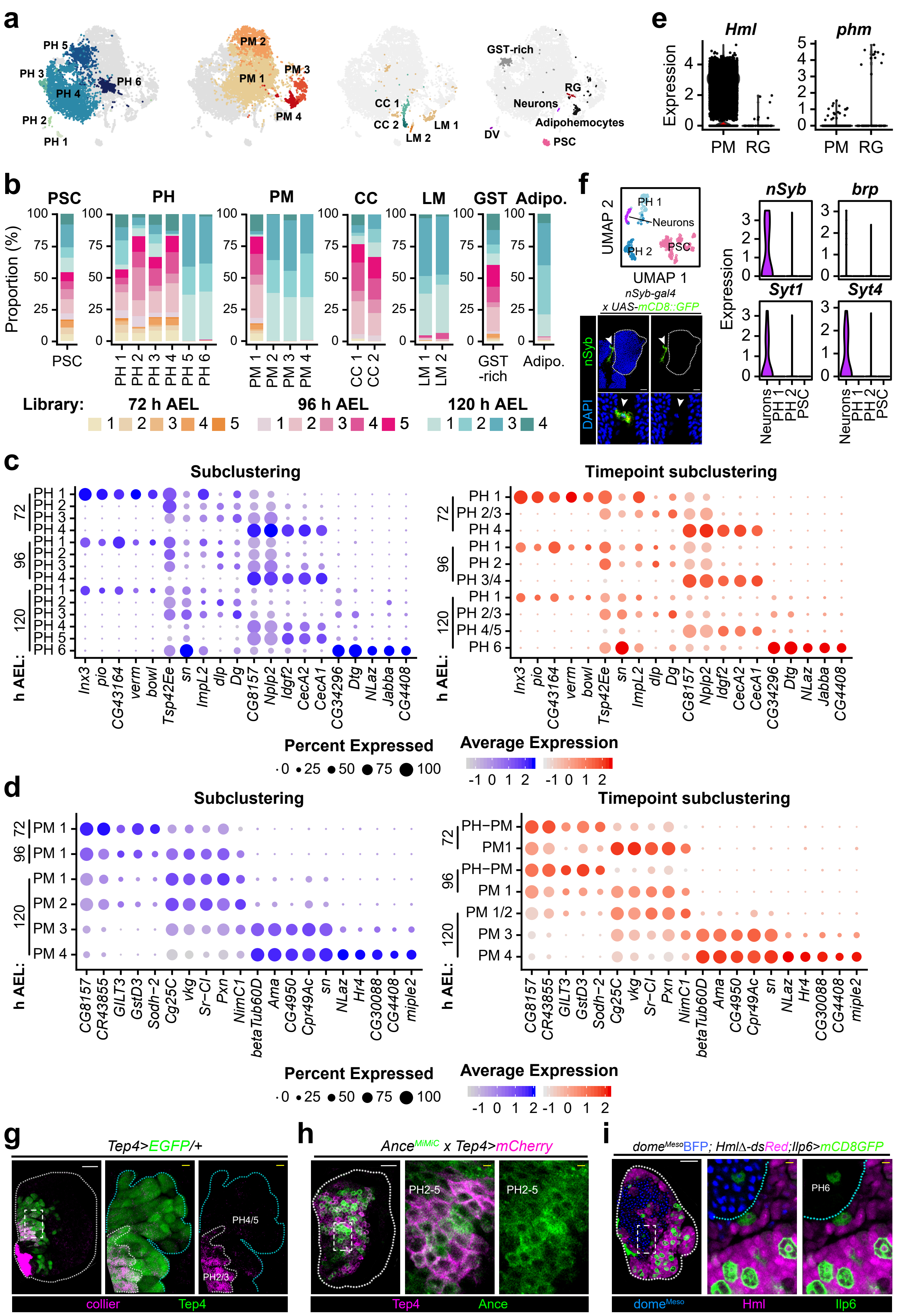

Supplementary Fig 2. Filtration of library-specific subclusters and *in vivo* validation of prohemocytes in the lymph gland

a. Subclusters exclusive to prohemocytes (PHs), exclusive plasmatocytes (PMs), crystal cells or lamellocytes (CCs or LMs), other hemocytes (GST-rich, adipohemocytes, and the PSC), and non-hematopoietic cells (neurons, dorsal vessel (DV), and ring gland (RG), in *t*-SNE plots.

b. Proportion of sequencing libraries per one subcluster found in major cell types. Orange, pink, or blue indicates 72, 96, or 120 h AEL, respectively. Color bar denotes each library.

c. Comparison of timepoint-combined (left) and -split (right) approaches in the subcluster level based on the top five highly enriched markers. For the timepoint-split approach, the subclustering of PH population was performed at each timepoint.

d. Comparison of timepoint-combined (left) and -split (right) approaches in the subcluster level based on the top five highly enriched markers. For the timepoint-split approach, the subclustering of PM population was performed at each timepoint.

e. Expression of *phm*, a ring gland marker. *Hml* is barely expressed in RG, and accordingly renamed as ring gland subcluster.

f. Identification of neuronal cells from subclustering analyses of PH1, PH2, and PSC (top left). Expression of neuronal marker genes—*nSyb*, *brp*, *Syt1*, and *Syt4*— in the prospective neuron subcluster (right). The presence of neuronal projections near the lymph gland (arrowhead; green, *nSyb*; blue, DAPI; *nSyb-gal4 UAS-mCD8::GFP*; bottom left).

g. Expression of PH2/PH3 and PH4/PH5 in the lymph gland. The dotted box indicates the region magnified at the right. Cells expressing col^{low} (magenta) partially overlap with *Tep4-gal4* (green) and represent PH2 and PH3. Outside col^{low} cells are putative PH4/PH5 that sustain *Tep4-gal4* only. The PSC exhibits col^{high}.

h. *Ance^{MiMiC}* (green) overlays well with *Tep4-gal4* (magenta) and together show prohemocytes except PH1. The dotted box indicates the region magnified at the right.

i. *Ilp6-gal4* (green) is detected in the PSC, crystal cells, and in *dome^{Meso+}* (blue) hemocytes. *Ilp6-gal4*-expressing crystal cells are limited to *Hml^{+}* (magenta) cortical zone and a few *Ilp6-gal4^{+}* cells are localized within *dome^{Meso+}* medullary zone. The *Ilp6-gal4/dome^{Meso}*-positive cells indicate the potential PH6. The dotted box indicates the region magnified at the right.

Scale bar is 30 μm. Lymph gland is demarcated by white dotted line. Arrowhead indicates a neuron. Magnified images of the left panel in **g-i** are shown at the middle and the right.

Supplementary Figure 3

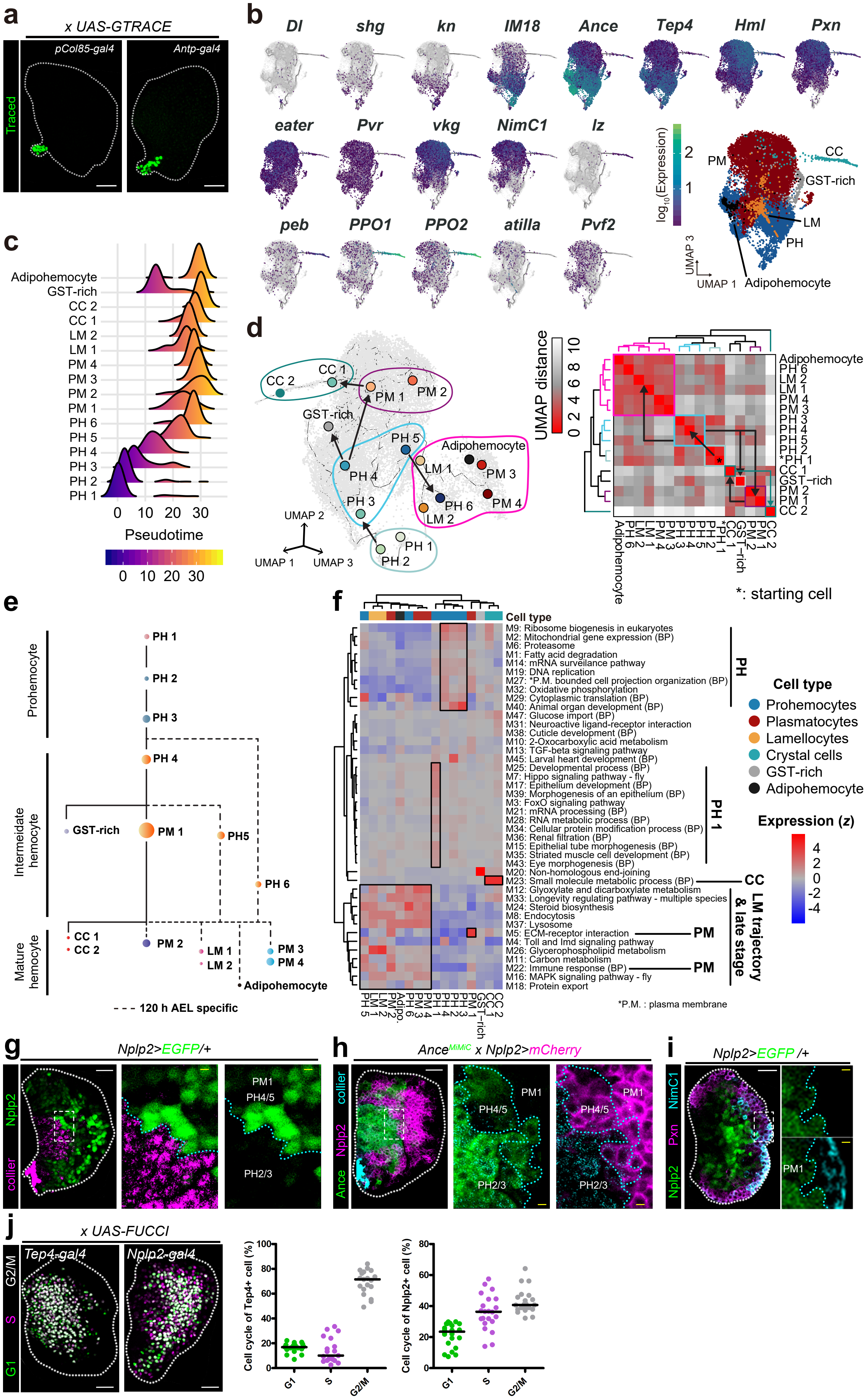

Supplementary Fig 3. Molecular features and distribution of subclusters along developmental pseudotime trajectories

a. PSC cells do not contribute to the rest of the lymph gland. Either *pCol85-gal4* or *Antp-gal4* is not traced beyond the PSC (green, traced; *pCol85-gal4 UAS-GTRACE* or *Antp-gal4 UAS-GTRACE*). Scale bar, 30 μm. Lymph glands are demarcated by white dotted lines.

b. Expression patterns of 18 marker genes—*Dl, shg, kn (col), IM18, Ance, Tep4, Hml, Pxn, eater, Pvr, vkg, NimC1, lz, peb, PPO1, PPO2, atilla, and Pvf2*—of major cell types in two-dimensional (UMAP 1 and 3) pseudotime trajectories. Distribution of cell types is shown at the bottom-right. Color bar denotes the $\log_{10}$-scaled level of gene expression. Grey color indicates no expression.

c. Relative densities of subclusters along pseudotime. PH1 and PH2 emerge at the earliest pseudotime. PH2-PH4 span through the mid-pseudotime while PH5 and PH6 peak at later timepoint. PM3-PM4, LM1, LM2, CC2, and adipohemocytes are the last cell types to differentiate.

d. Distribution of subclusters in three-dimensional pseudotime trajectory (left), and clustering of distances between subclusters (right). Subclusters are grouped according to distances and directions of differentiation (left). PH1 and PH2 (white box) are the earliest subclusters among prohemocytes. PH6, PM3, PM4, adipohemocytes, LM1, and LM2 (pink box) form a separate group. PM1 is diverged to CC1 or PM2 (purple box). Color bar denotes the UMAP distance (right).

e. Simplified stick-and-ball presentation of the trajectory. Dotted lines show lineages that appear only at 120 h AEL. Size of the ball represents subcluster proportion.

f. Heatmap showing the z-transformed mean expression of gene modules of subclusters. Gene modules clustered in a specific cell type are indicated in black boxes. The column annotation indicates major cell types used in the analysis.

g. *Nplp2-gal4* (green; *Nplp2-gal4 UAS-EGFP*) and col^{low} prohemocytes (magenta) are mutually exclusive. col^{low}-positive inner core prohemocytes correspond with PH2 and PH3 and *Nplp2-gal4*-expressing cells give rise subsequent to PH2/PH3. Magnified images are shown at the right of each panel. Cyan dotted line demarcates the margins of *Nplp2*.

h. Inner demarcation of *Nplp2-gal4* cells (magenta; *Nplp2-gal4 UAS-mCherry; Ance^{MiMiC}*) overlays with *Ance* (green) while separable from col^{low} (cyan). Magnified images are shown at the right of each panel. Cyan dotted line demarcates the margins of *Ance* and *Nplp2*.

i. *Nplp2-gal4* (green) does not overlap with a mature plasmatocyte marker, NimC1 (cyan) (right; *Nplp2-gal4 UAS-EGFP*). Magnified images are shown at the right of each panel. Cyan dotted line demarcates the margins of *Nplp2*.

j. Cell cycle profiles of *Nplp2-gal4*$^{+}$ cells differ from that of *Tep4-gal4*. The majority of *Tep4*$^{+}$ (*Tep4-gal4 UAS-FUCCI*) cells display G2/M (white), and a minor portion expresses G1 (green) or S (magenta) (left). *Nplp2-gal4 UAS-FUCCI* shows higher levels of S (magenta) and lower G2/M (white) compared to *Tep4-gal4* (right). Graph indicates quantitation of the number of FUCCI cell cycle markers in one lymph gland lobe. *Tep4-gal4 UAS-FUCCI* (*n*=19), *Nplp2-gal4 UAS-FUCCI* (*n*=23). Lymph glands were analyzed from three independent experiments.

White scale bar, 30 μm. Yellow scale bar, 3 μm. Lymph glands are demarcated by white dotted lines.

Supplementary Figure 4

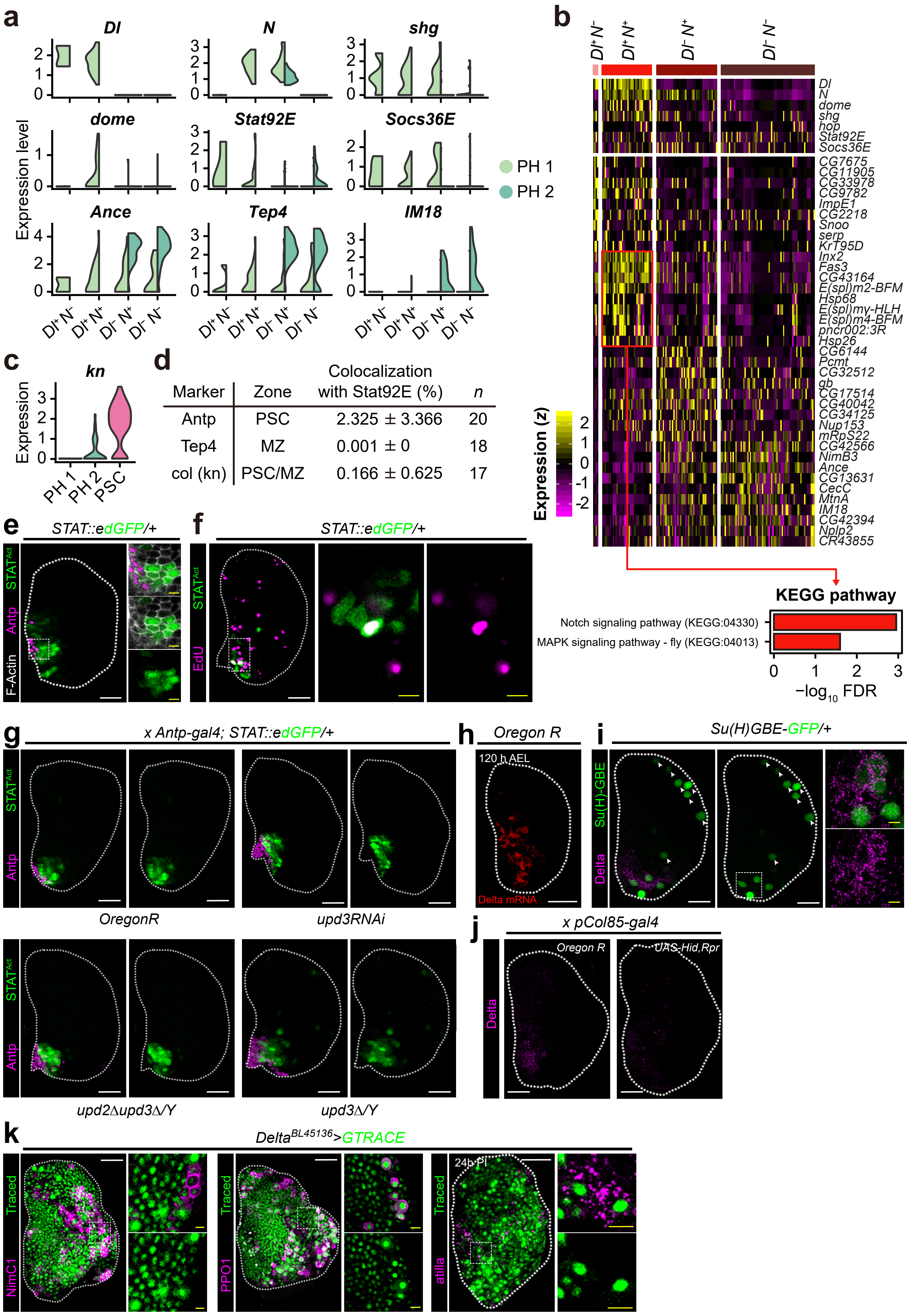

Supplementary Fig 4. PH1 cells express Dl and JAK/STAT

a. Expression of PH1- or prohemocyte marker genes in *Dl^+N^-, Dl^+N^+, Dl^-N^+,* or *Dl^-N^-* PH1. Expression values of PH1 (light green) and PH2 (green) are shown separately.

b. Marker genes classified by the differential expression of *Dl* and *N* in PH1 and PH2. *Dl^+N^-* cells exhibit high levels of *Dl*, *shg*, *STAT92E*, and *Socs36E*. *Dl^+N^+* cells show gradual decrease or increase in *Dl* or *N* and express *Inx2*, *Fas3*, CG43164, and *E(spl)m2-BFM*. *Dl^-N^+* cells display *CG6144*, *Pcmt*, and *CG32512* along with high *N*. *Dl^-N^-* cells are distinguishable from other cell types and express high *Ance*. *Dl^+N^+* cells show enriched MAPK and Notch signaling pathways based on KEGG pathway analysis. Color bar indicates the level of scaled gene expression.

c. Violin plots indicating differential levels of *kn* (*col*) in PH1, PH2 and in the PSC.

d. Quantitation of co-localization of *Stat92E::edGFP* and representative marker genes, *Antp*, *Tep4*, or *col*. *Antp*$^+$ PSC, *Tep4*$^+$ medullary zone or *col*$^+$ PSC/medullary zone cells do not show significant overlap with *Stat92E::edGFP*. *n* indicates the number of samples. Lymph glands were analyzed from three independent experiments.

e. *$STAT92E^{Act}$* PH1 cells (green) physically interact with Antp$^+$ (magenta) PSC cells (left). When visualized with F-actin (white), Antp$^+$ cells and *$STAT92E^{Act}$* cells share F-actin membrane but do not co-localize (right).

f. EdU incorporation in *$STAT92E^{Act}$* PH1 cells (green). *$STAT92E^{Act}$* PH1 cells indicate EdU expression (magenta) along with EdU$^+$ proliferating cells adjacent to *$STAT92E^{Act}$* cells. The dotted box indicates the region magnified at the right.

g. *upd3* in the PSC is dispensable for the maintenance of *STAT92E*^{Act}. Compared to the wild type (upper left; *Antp-gal4/+*), PSC-specific reduction of *upd3* (upper right; *STAT92E::edGFP*; *Antp-gal4 UAS-upd3 RNAi*), loss of *upd2* and *upd3* (bottom left; *upd2Δupd3Δ/Y; STAT92E::edGFP*)or *upd3* alone (bottom right; *upd3Δ/Y; STAT92E::edGFP*) does not alter the expression of *STAT92E* reporter (green). The presence of PSC is indicated in magenta.

h. Fluorescent *in situ* hybridization of *Dl* mRNA (red) in the lymph gland at 120 h AEL. *Dl* is localized in the medioposterior region of the lymph gland.

i. *Su(H)-GBE* (green; *Su(H)-GBE-GFP*), a Notch reporter, is expressed adjacent to Dl^{+} PH1 (magenta). *Su(H)-GBE* is also detected in the crystal cells (arrowhead). Magnified images of Dl^{+} cells are shown at the right.

j. Genetic ablation of the PSC (*pCol85-gal4 UAS-Hid, Rpr*) attenuates Dl (magenta) expression in the lymph gland (right) compared to wild types (left).

k. Lineage tracing of *Dl*$^{+}$ cells (green, traced). *Delta*^{BL45136}*-gal4 UAS-GTRACE* gives rise to all three classes of hemocytes including NimC1-expressing plasmatocytes (left; magenta), PPO1^{+} crystal cells (middle; magenta), and atilla^{+} lamellocytes (right; magenta). Box indicates the magnified view.

White scale bar, 30 μm. Yellow scale bar, 3 μm. Lymph glands are demarcated by white dotted lines.

Supplementary Figure 5

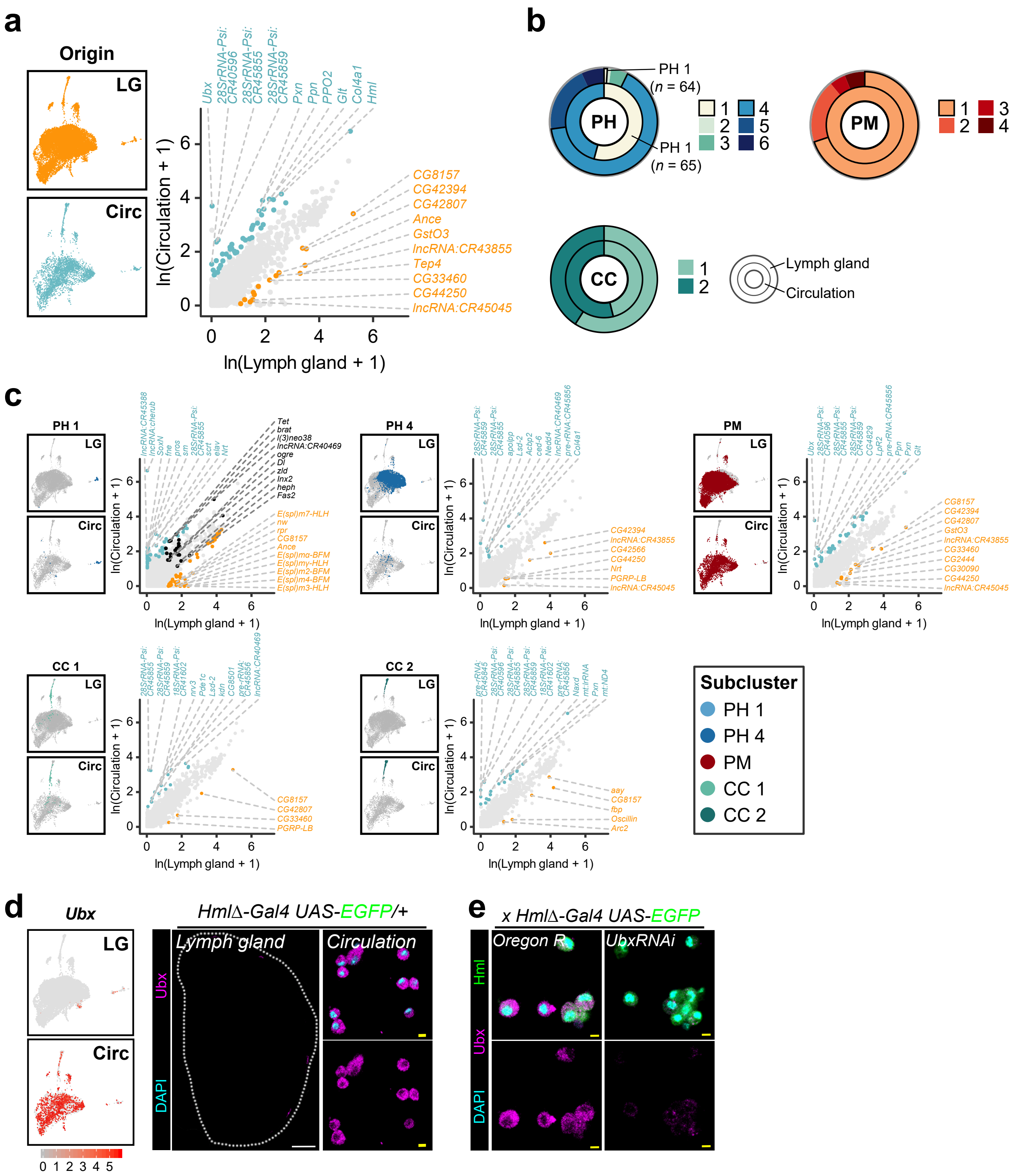

Supplementary Fig 5. Lineage-specific cell types and marker genes for the lymph gland- or the embryonically derived hemocytes

a. Comparison of gene expressions between the lymph gland and circulating hemocytes. Top ranked lymph gland- (right) or circulation-specific (top) differentially expressed genes are indicated in colored gene names (orange, upregulated in lymph glands; cyan, upregulated in circulation).

b. Pie charts showing the relative proportion of subclusters in the lymph gland and in circulation datasets. The outer graph indicates proportions of the lymph gland and the inner graph shows those of the circulation dataset. Demarcated subclusters indicate those found in circulation. Cell count for each subcluster is listed in Supplementary Table 6.

c. Comparison of gene expressions between the lymph gland and in circulation. PH1, PH4, PM, CC1, and CC2. Top ranked lymph gland- (right) or circulation-specific (top) differentially expressed genes are indicated in colored gene names (orange, upregulated in lymph glands; cyan, upregulated in circulation).

d. Expression of *Ubx* is exclusive in circulating plasmatocytes. Comparison of *Ubx* levels between the lymph gland and in circulation (left). The lymph gland does not show *Ubx* expression (cyan, DAPI; left). Circulating plasmatocytes display *Ubx* (cyan, DAPI; magenta, Ubx; right) (*HmlΔ-gal4 UAS-EGFP/+* control). White scale bar indicates 30 μm. Yellow scale bar indicates 3 μm. White dotted line demarcates the lymph gland.

e. Verification of *Ubx* expression in circulating hemocytes. Expression levels of *Ubx* in circulating plasmatocytes are significantly reduced when RNAi against *Ubx* is expressed

(green, Hml; cyan, DAPI; magenta, Ubx) (*Hml-gal4 UAS-EGFP UAS-UbxRNAi*). Yellow scale bar indicates 3 μm.

Supplementary Table 1. Basic statistics of lymph grand scRNA-seq libraries

Timepoint	# of cells	UMI median	Gene median
72 h AEL lib1	954	9,242	1,721.5
72 h AEL lib2	424	7,879	1,636
72 h AEL lib3	217	10,142	1,726
72 h AEL lib4	589	8,858	1,542
72 h AEL lib5	137	15,937	2,570
**72 h AEL total**	**2,321**	**9,297**	**1,704**
96 h AEL lib1	603	8,210	1,779
96 h AEL lib2	3,478	4,564	1,204
96 h AEL lib3	1,095	8,134	1,699
96 h AEL lib4	2,332	5,597	1,396
96 h AEL lib5	1,892	5,679	1,408
**96 h AEL total**	**9,400**	**5,507**	**1,373**
120 h AEL lib1	2,449	7,456	1,668
120 h AEL lib2	1,901	9,304	1,843
120 h AEL lib3	4,221	7,193	1,576
120 h AEL lib4	2,353	4,406	1,167
**120 h AEL total**	**10,924**	**6,751**	**1,537**
**LG total**	**22,645**	**6,361**	**1,477**

Supplementary Table 2. Basic statistics of scRNA-seq libraries from wasp-infested LG

Timepoint	# of cells	UMI median	Gene median
24 h PI lib1	2,109	7,701	1,760
24 h PI lib2	1,809	8,039	1,872
24 h PI lib3	4,532	4,761	1,337
24 h PI lib4	1,708	3,907	1,189.5
**24 h PI total**	**10,158**	**5,561.5**	**1,470.5**

Supplementary Table 3. Basic statistics of scRNA-seq libraries from circulation hemocytes

Timepoint	# of cells	UMI median	Gene median
Circ 96 h AEL lib1	433	5,434	1,581
Circ 96 h AEL lib2	153	5,859	1,694
Circ 96 h AEL lib3	409	4,282	1,372
**Circ 96 h AEL total**	**995**	**4,979**	**1,489**
Circ 120 h AEL lib1	531	4,616	1,412
Circ 120 h AEL lib2	367	6,088	1,692
Circ 120 h AEL lib3	473	4,312	1,342
**Circ 120 h AEL total**	**1,494**	**4,810**	**1,462**

Supplementary Table 4. Cell count of lymph gland subclusters

Subclusters	72 h AEL	96 h AEL	120 h AEL	LG total
**PSC**	32	70	87	189
**PH1**	14	30	34	78
**PH2**	9	58	14	81
**PH3**	78	197	139	414
**PH4**	989	3,374	893	5,256
**PH5**	0	0	1,321	1,321
**PH6**	2	3	445	450
**PM1**	1,065	5,259	1,340	7,664
**PM2**	0	1	1,811	1,812
**PM3**	0	0	457	457
**PM4**	2	3	575	580
**LM1**	2	10	265	277
**LM2**	0	4	59	63
**CC1**	3	124	39	166
**CC2**	0	75	38	113
**GST-rich**	13	126	94	233
**Adipohemocyte**	1	5	172	178
**Total**	**2,210**	**9,339**	**7,783**	**19,332**

Supplementary Table 5. Cell count of wasp infested lymph grand subclusters

Subclusters	Infested LG	Normal LG
**PSC**	82	70
**PH1**	16	30
**PH2**	21	58
**PH3**	141	197
**PH4**	4,579	3,374
**PM1**	4,674	5,259
**LM1**	208	10
**LM2**	168	4
**CC1**	11	124
**CC2**	0	75
**GST-rich**	258	126
**Total**	**10,158**	**9,327**

*Following minor populations found only in normal LG were excluded in the analysis
: PH 6, PM 2, 4, Adipohemocyte

Supplementary Table 6. Cell count of pepripheral and lymph grand subclusters

Subclusters	Circ 96 h AEL	Circ 120 h AEL	Circulating total	LG 96 h AEL	LG 120 h AEL	LG total
**PSC**	0	0	0	70	87	157
**PH1**	30	35	65	30	34	64
**PH2**	0	0	0	58	14	72
**PH3**	0	0	0	197	139	336
**PH4**	0	55	55	3,374	893	4,267
**PH5**	0	0	0	0	1,321	1,321
**PH6**	0	0	0	3	445	448
**PM1**	879	2,205	3,084	5,259	1,340	6,599
**PM2**	0	0	0	1	1,811	1,812
**PM3**	0	0	0	0	457	457
**PM4**	0	0	0	3	575	578
**LM1**	0	0	0	10	265	275
**LM2**	0	0	0	4	59	63
**CC1**	52	37	89	124	39	163
**CC2**	34	70	104	75	38	113
**GST-rich**	0	0	0	126	94	220
**Adipohemocyte**	0	0	0	5	172	177
**Total**	**995**	**2,402**	**3,397**	**9,339**	**7,783**	**17,122**

Supplementary Table 7. SABER-FISH probe list

Probe Name	Probe Sequence
**Catalytic hairpin**	ACATCATCATGGGCCTTTTGGCCCATGATGATGTATGATGATG/3InvdT/
**Image seq**	/Biotin/TTATGATGATGTATGATGATGT
**Clean.G**	CCCCGAAAGTGGCCTCGGGCCTTTTGGCCCGAGGCCACTTTCG
**CG3397**	TGACTCCGATGAATCGAGCCTTTCCCGCTTTCATCATCAT
	CCATCCAGTTGCATTGTATAGTACATGGCCAGCTTTCATCATCAT
**CG18547**	CTTTCGCGCGTCTTCTTGGCACTAAAGTCTTTCATCATCAT
	TCGTGTAGTACATGGCCAGCTTGCCCTTTCATCATCAT
**Lsd-2**	GCGCCTTTACCTCAAGCTTATCGATGCCTTTCATCATCAT
	GCCTTCTGCTGACCAGCTGCCTTGAACTTTCATCATCAT
**Sirup**	ATTAGCTTCTCGGTGCGCGTCTTTGGTTTCATCATCAT
	ATACGGATTGGTCTGATTGGGCCAGGGTTTCATCATCAT
