# Supplementary material for: Single-cell transcriptome maps of myeloid blood cell lineages in Drosophila
Source: Nat Commun. 2020 Sep 8;11:4483. doi: 10.1038/s41467-020-18135-y (PMC7479620; doi:10.1038/s41467-020-18135-y)
Supplement: Supplementary file 3 — Description of Additional Supplementary Files [file 41467_2020_18135_MOESM3_ESM.pdf]

Title: Supplementary Movie 1
Description: Three-dimensional trajectory landscape

Title: Supplementary Data 1
Description: Signature genes for hemocytes and non-hematopoietic cell types using Wilcoxon Rank-Sum test

Title: Supplementary Data 2
Description: Signature genes for subclusters using Wilcoxon Rank-Sum test

Title: Supplementary Data 3
Description: Putative Drosophila markers screened in this study

Title: Supplementary Data 4
Description: Putative markers for embryonic lymph gland hemocytes

Title: Supplementary Data 5
Description: Putative markers for each subcluster

Title: Supplementary Data 6
Description: Gene modules of the lymph gland trajectory analysis in Supplementary Fig. 3f

Title: Supplementary Data 7
Description: Gene modules of the lamellocyte subtrajectory analysis in Fig. 7d

Title: Supplementary Data 8
Description: Lineage-specific signature genes for subclusters
