# Supplementary material for: Single-cell transcriptome maps of myeloid blood cell lineages in Drosophila
Source: Nat Commun. 2020 Sep 8;11:4483. doi: 10.1038/s41467-020-18135-y (PMC7479620; doi:10.1038/s41467-020-18135-y)
Supplement: Supplementary file 13 — Reporting Summary [file 41467_2020_18135_MOESM13_ESM.pdf]

nature research

Corresponding author(s): Jiwon Shim, Jin-Wu Nam

Last updated by author(s): Jul 26, 2020

Reporting Summary

Nature Research wishes to improve the reproducibility of the work that we publish. This form provides structure for consistency and transparency in reporting. For further information on Nature Research policies, see our Editorial Policies and the Editorial Policy Checklist.

Statistics

For all statistical analyses, confirm that the following items are present in the figure legend, table legend, main text, or Methods section.

n/a	Confirmed	
☐	☒	The exact sample size (*n*) for each experimental group/condition, given as a discrete number and unit of measurement
☐	☒	A statement on whether measurements were taken from distinct samples or whether the same sample was measured repeatedly
☐	☒	The statistical test(s) used AND whether they are one- or two-sided<br>*Only common tests should be described solely by name; describe more complex techniques in the Methods section.*
☐	☒	A description of all covariates tested
☐	☒	A description of any assumptions or corrections, such as tests of normality and adjustment for multiple comparisons
☐	☒	A full description of the statistical parameters including central tendency (e.g. means) or other basic estimates (e.g. regression coefficient) AND variation (e.g. standard deviation) or associated estimates of uncertainty (e.g. confidence intervals)
☐	☒	For null hypothesis testing, the test statistic (e.g. *F*, *t*, *r*) with confidence intervals, effect sizes, degrees of freedom and *P* value noted<br>*Give P values as exact values whenever suitable.*
☒	☐	For Bayesian analysis, information on the choice of priors and Markov chain Monte Carlo settings
☐	☒	For hierarchical and complex designs, identification of the appropriate level for tests and full reporting of outcomes
☐	☒	Estimates of effect sizes (e.g. Cohen's *d*, Pearson's *r*), indicating how they were calculated

Our web collection on statistics for biologists contains articles on many of the points above.

Software and code

Policy information about availability of computer code

Data collection	Prism ver.8, Excel, R, IMARIS ver8.3, SPSS ver.26, Seurat 3, Monocle 2, Monocle 3, and Python
Data analysis	All the biological experiments were normalized with SPSS. If samples do not follow the normal distribution, data were analyzed by the Mann-Whitney test. In-house R and Python codes that were implemented in this study are available on GitHub (https://github.com/sangho1130/Dmel_Dropseq). Flybase was used for the reference database.

For manuscripts utilizing custom algorithms or software that are central to the research but not yet described in published literature, software must be made available to editors and reviewers. We strongly encourage code deposition in a community repository (e.g. GitHub). See the Nature Research guidelines for submitting code & software for further information.

Data

Policy information about availability of data

All manuscripts must include a data availability statement. This statement should provide the following information, where applicable:

- Accession codes, unique identifiers, or web links for publicly available datasets
- A list of figures that have associated raw data
- A description of any restrictions on data availability

In-house R and Python codes that were implemented in this study are available on GitHub (https://github.com/sangho1130/Dmel_Dropseq). Raw scRNA-seq and bulk RNA-seq reads are available through the NCBI Gene Expression Omnibus (GEO) (GSE141275). Processed scRNA-seq data sets can be mined through a web-tool (http://big2.hanyang.ac.kr/flyscrna) that allows users to explore genes and cell types of interest. Flybase was used for the reference database.

April 2020

Field-specific reporting

Please select the one below that is the best fit for your research. If you are not sure, read the appropriate sections before making your selection.

- [x] Life sciences
- [ ] Behavioural & social sciences
- [ ] Ecological, evolutionary & environmental sciences

For a reference copy of the document with all sections, see nature.com/documents/nr-reporting-summary-flat.pdf

Life sciences study design

All studies must disclose on these points even when the disclosure is negative.

Sample size	For the scRNA-seq experiments, the sample size was chosen by cell numbers. More than one pair of lymph gland primary lobes were covered with proper cell number. For lymph gland experiments, a minimum of three independent experiments was conducted.
Data exclusions	Outliers were excluded in the datasets where scRNA-seq data and Bulk RNA seq data were compared. Except for this, all data sets were included in this manuscript.
Replication	A minimum of three independent experiments was performed.
Randomization	When performed the experiments, samples were randomly chosen for experiments.
Blinding	A person who analyzed data (Sang-Ho Yoon) is different from one who performed experiments (Bumsik Cho and others). Sample preparation was performed by Bumsik Cho and running the Drop-seq machine was performed by Sue Young Oh.

Reporting for specific materials, systems and methods

We require information from authors about some types of materials, experimental systems and methods used in many studies. Here, indicate whether each material, system or method listed is relevant to your study. If you are not sure if a list item applies to your research, read the appropriate section before selecting a response.

Materials & experimental systems

n/a	Involved in the study
[ ]	[x] Antibodies
[x]	[ ] Eukaryotic cell lines
[x]	[ ] Palaeontology and archaeology
[ ]	[x] Animals and other organisms
[x]	[ ] Human research participants
[x]	[ ] Clinical data
[x]	[ ] Dual use research of concern

Methods

n/a	Involved in the study
[x]	[ ] ChIP-seq
[x]	[ ] Flow cytometry
[x]	[ ] MRI-based neuroimaging

Antibodies

Antibodies used	lozenge (DSHB, 1:10, Mouse), Antp (4C3, DSHB, 1:10, Mouse), Dl (C594.9B, DSHB, 1:10, Mouse), L1 (I.Ando, 1:100, Mouse), col (M.Crozatier, 1:400, Mouse), Ubx (FP3.38, DSHB, 1:10, Mouse), nc82 (DSHB, 1:10, Mouse), NimC1 (I.Ando, 1:100), Pxn(1:2000, Rabbit), GFP (Sigma Aldrich; G6539; 1:2,000, Mouse) and F-actin (ThermoFisher; A34055, 1:100). Cy3-, FITC- or Alexa Fluor 647-conjugated secondary antibody(115-165-166, 711-165-152, 115-095-062, 711-095-152, 715-605-151), DIG-biotin antibody (Jackson Immunoresearch; 200-062-156).
Validation	Most of the antibodies used in this study are previously validated. Expression of the Ubx antibody (FP3.38, DSHB, 1:10) in hemocytes was first shown in this study. To validate the Ubx antibody, we used RNAi against Ubx and showed that Ubx expression in the hemocyte is decreased.

Animals and other organisms

Policy information about studies involving animals; ARRIVE guidelines recommended for reporting animal research

Laboratory animals	Drosophila melanogaster
Wild animals	No wild animal was used in this study.
Field-collected samples	No field collected sample was used in this study.
Ethics oversight	None

Note that full information on the approval of the study protocol must also be provided in the manuscript.
